# Effect of osteoporosis induced by ovariectomy on vertebral bone defect/fracture in rat

**DOI:** 10.18632/oncotarget.20611

**Published:** 2017-09-01

**Authors:** Geng-Yang Shen, Hui Ren, Jing-Jing Tang, Ting Qiu, Zhi-Da Zhang, Wen-Hua Zhao, Xiang Yu, Jin-Jing Huang, De Liang, Zhen-Song Yao, Zhi-Dong Yang, Xiao-Bing Jiang

**Affiliations:** ^1^ Guangzhou University of Chinese Medicine, Guangzhou, China; ^2^ Department of Spinal Surgery, The First Affiliated Hospital of Guangzhou University of Chinese Medicine, Guangzhou, China; ^3^ Laboratory Affiliated to National Key Discipline of Orthopaedic and Traumatology of Chinese Medicine, Guangzhou University of Chinese Medicine, Guangzhou, China

**Keywords:** osteoporosis, vertebral bone defect/fracture, ovariectomy, rat, Pathology Section

## Abstract

Osteoporotic vertebral fracture (OVF) is a worldwide health concern and lacks sufficient basic studies. Suitable animal models should be the foundation for basic study and treatment of OVF. There have been few studies on the development of animal models of osteoporotic vertebral bone defects. OVF models using various animal species should be developed to evaluate the therapeutic strategy in preclinical testing. We developed an OVF model in rats. Rat osteoporosis was induced by ovariectomy (OVX), and 3 months after OVX, a 3 mm diameter hemispheric vertebral bone defect was developed in lumbar vertebra 6 (L6). Sagittal plain X-rays of the rats, their bone quantity, bone microarchitecture, and histomorphology were analyzed: 3 months after OVX, rats showed significantly lower bone quantity, relative bone volume, and total volume bone mineral density. After the vertebral bone defect had developed for 16 weeks, no significant indication of self-healing could be observed from the sagittal plain X-rays, three-dimensional images, and histomorphology. These results indicate that the rat model of osteoporotic vertebral bone defect, induced by OVX and a 3 mm diameter hemispheric vertebral bone defect, can sufficiently mimic OVF patients in clinic and provide a sound basis for subsequent studies.

## INTRODUCTION

Osteoporotic vertebral fracture (OVF), one of the most common complications of osteoporosis (OP), is associated with considerable disability and expense and increased risk of mortality [1, 2]; they are often asymptomatic when they first occur, with many cases going undiagnosed for a considerable time [3-5]. Approximately 50% of 65-year-old postmenopausal white or Asian women will experience an osteoporotic fracture, and clinicians have to deal with an estimated 1-4 million OVFs with pain, disability, and inferior quality of life every year worldwide [6, 7, 8]. Therefore, more basic studies that focus on OVF in detail are necessary. However, basic studies of OVF are limited because OVF animal models have yet to be properly established, as only a few studies of such models are available in the literatures [9, 10].

Recently, a small number of studies have demonstrated that several vertebral bone defect animal models, such as rat, ovine, canine, and swine models, can be developed to describe the characteristics of vertebral fracture (VF) [11]. However, basic studies of the development of animal models of osteoporotic vertebral bone defects that can sufficiently mimic OVF patients in clinical settings remain limited. The OVF animal models that have been reported to date are limited to rabbits and sheep [9, 10]. However, ovariectomy (OVX), which is performed to induce OP, has a low modeling success rate in rabbits [12], and bone mineral density (BMD), blood biochemical index, and bone tissue morphology in sheep fluctuate seasonally [13]. Moreover, the US Food and Drug Administration (FDA) established guidelines in 1994 indicating that novel protocols against OP should be evaluated using at least two animal species in preclinical testing. In response to this need for an effective animal model for the study of therapeutic strategies for the repair of OVF, such as biomaterial interventions, we developed an osteoporotic vertebral bone defect model in rats. We also evaluated its usefulness.

Rats underwent OVX and then surgery to create a vertebral bone defect of an appropriate size using uniform specification drills. Then these rats were compared to untreated animals. Lumbar spine BMD and bone mineral content (BMC) were measured to determine bone quantity using dual-energy X-ray absorptiometry (DXA). Sagittal plain X-rays of the rats were obtained to show the sites of vertebral bone defects and assess the self-healing status of bone defects at different time points. Bone microarchitecture, detected by micro computed tomography (micro-CT), was used to describe bone quality. Hematoxylin and eosin (HE) staining and Safranin-fast green staining were applied to observe the histomorphology of the target vertebrae.

## RESULTS

### Effects on BMD and BMC

**Figure 1:**
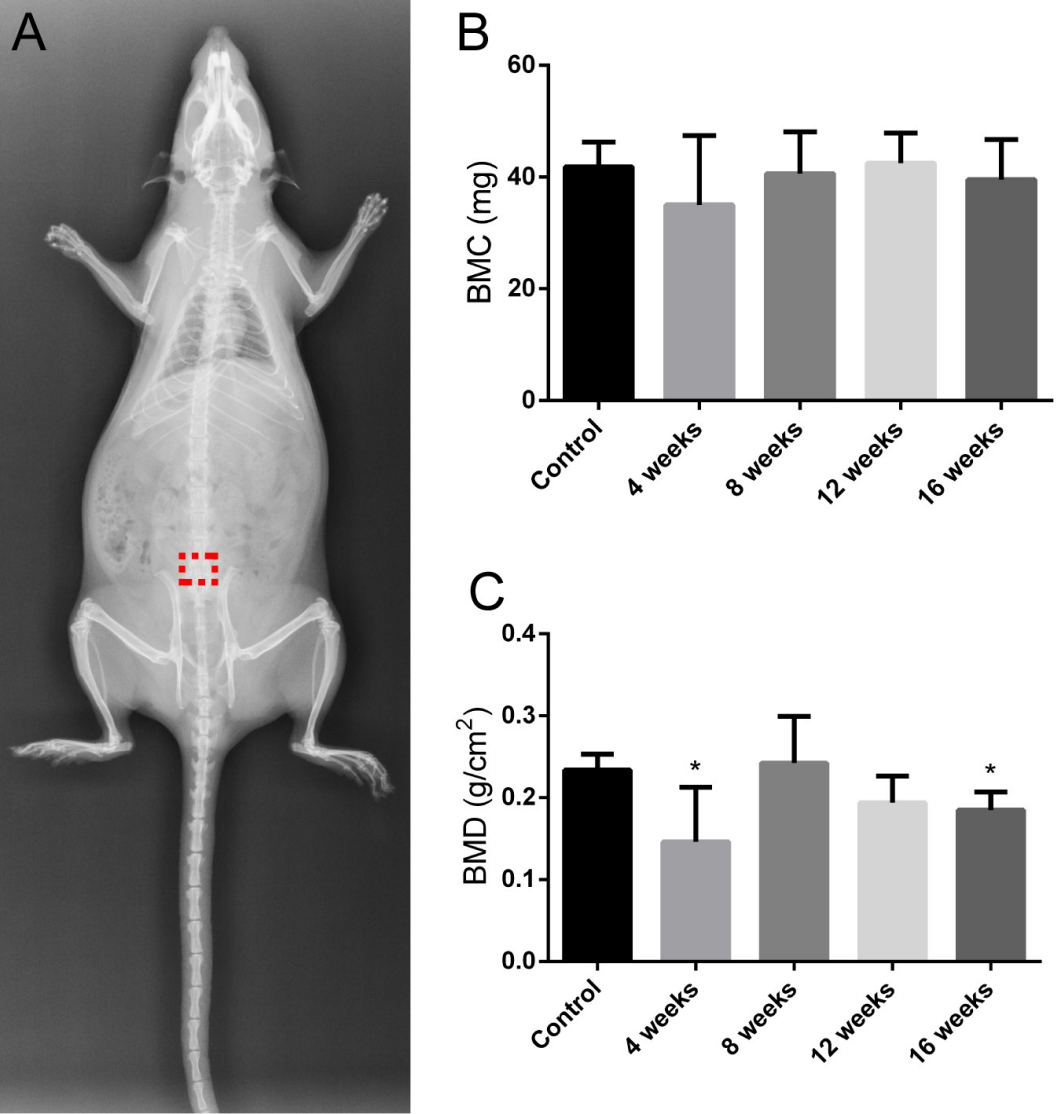
X-ray image of the rat in the prone position in the control group The red dotted box represents bone defect site **A.**The changes in BMC and BMD of L1-3. Values are the means ± SD. ^*^P < 0.05 *vs* the control group (*n* = 6) **B.** and **C.**

To verify the OP rat model, the levels of BMD and BMC were detected by DXA. Compared to the control group, the model group showed a significantly lower BMD at weeks 4 and 16 (*P* < 0.05), and a lower BMC at week 4. This difference was not, however, statistically significant. In the model group, no significant differences were observed in BMC and BMD among the different time points (Figure 1).

### Changes to the vertebral bone defect model

**Figure 2:**
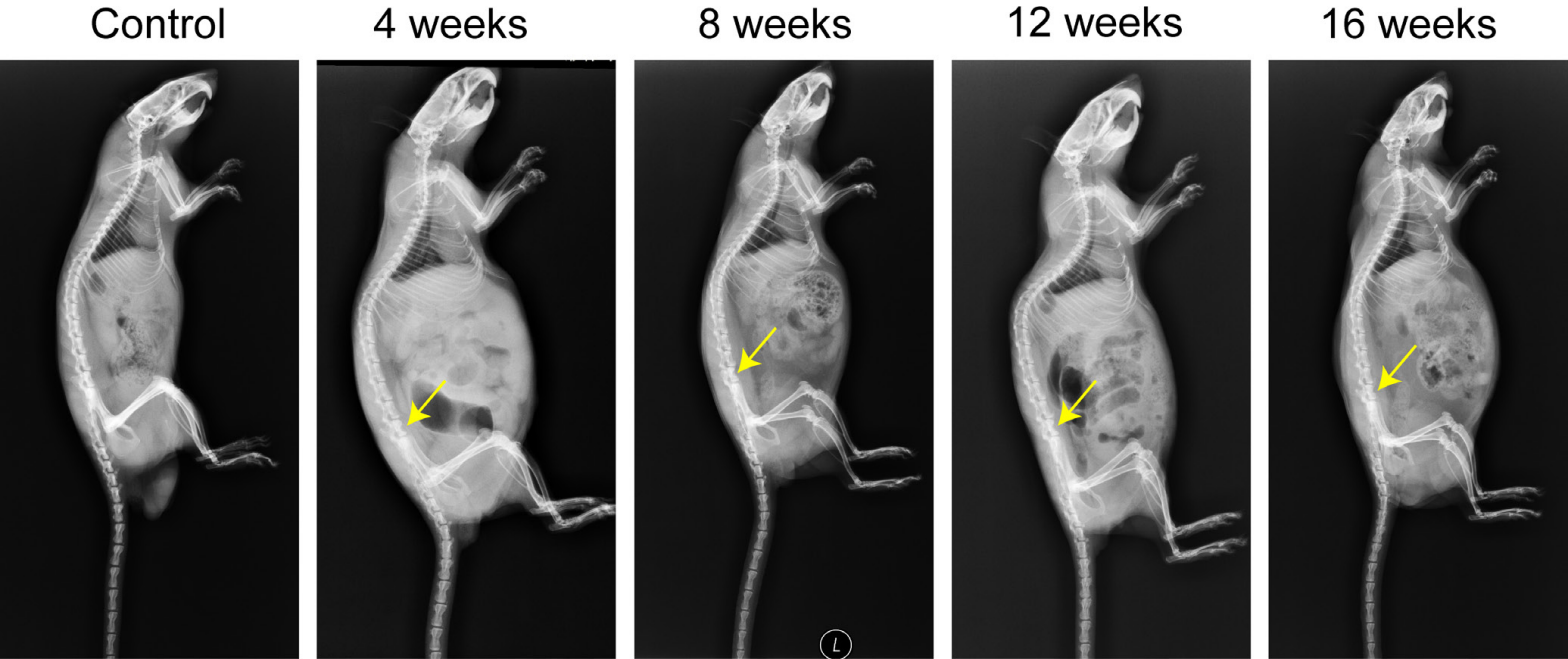
Assessment of the self-healing condition of bone defect of L6 The yellow arrows represent bone defect sites.

**Figure 3:**
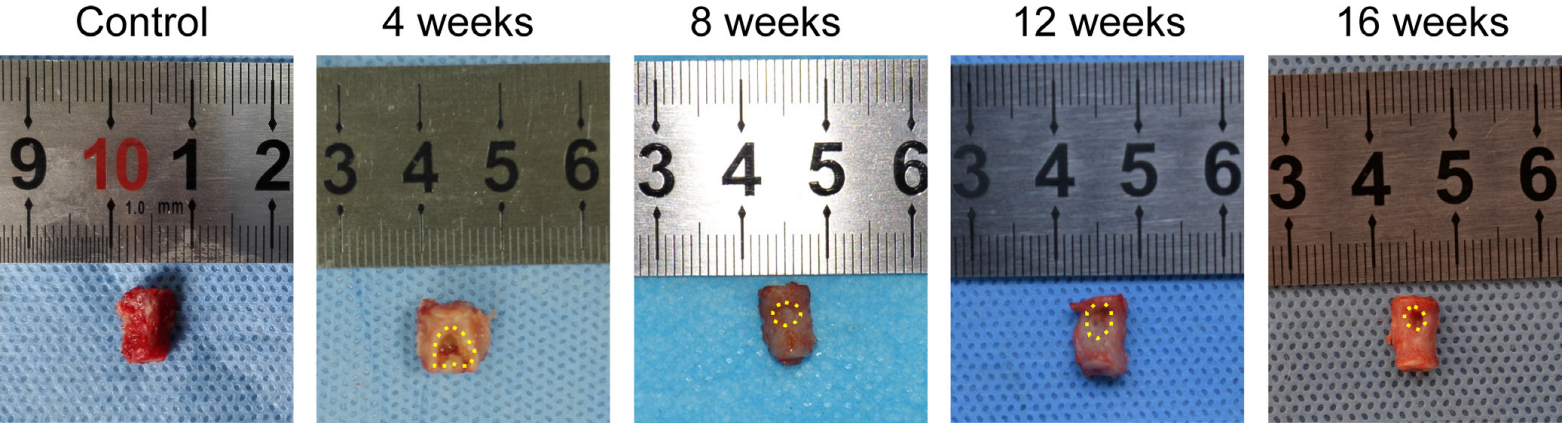
L6 samples *in vitro* at M0 and weeks 4, 8, 12, and 16 after OVX The bone defect sites are encircled by the small yellow dots.

The bone defect and healing condition were verified by sagittal plain X-rays (Figure 2) and *ex vivo* naked-eye observations (Figure 3). Compared to the control group, the model group showed a significant bone defect in lumbar vertebra 6 (L6) at different time points. Furthermore, in the model group, no significant indication of self-healing in the bone defect site was observed among the different time points.

### Effects on bone microarchitecture

**Figure 4:**
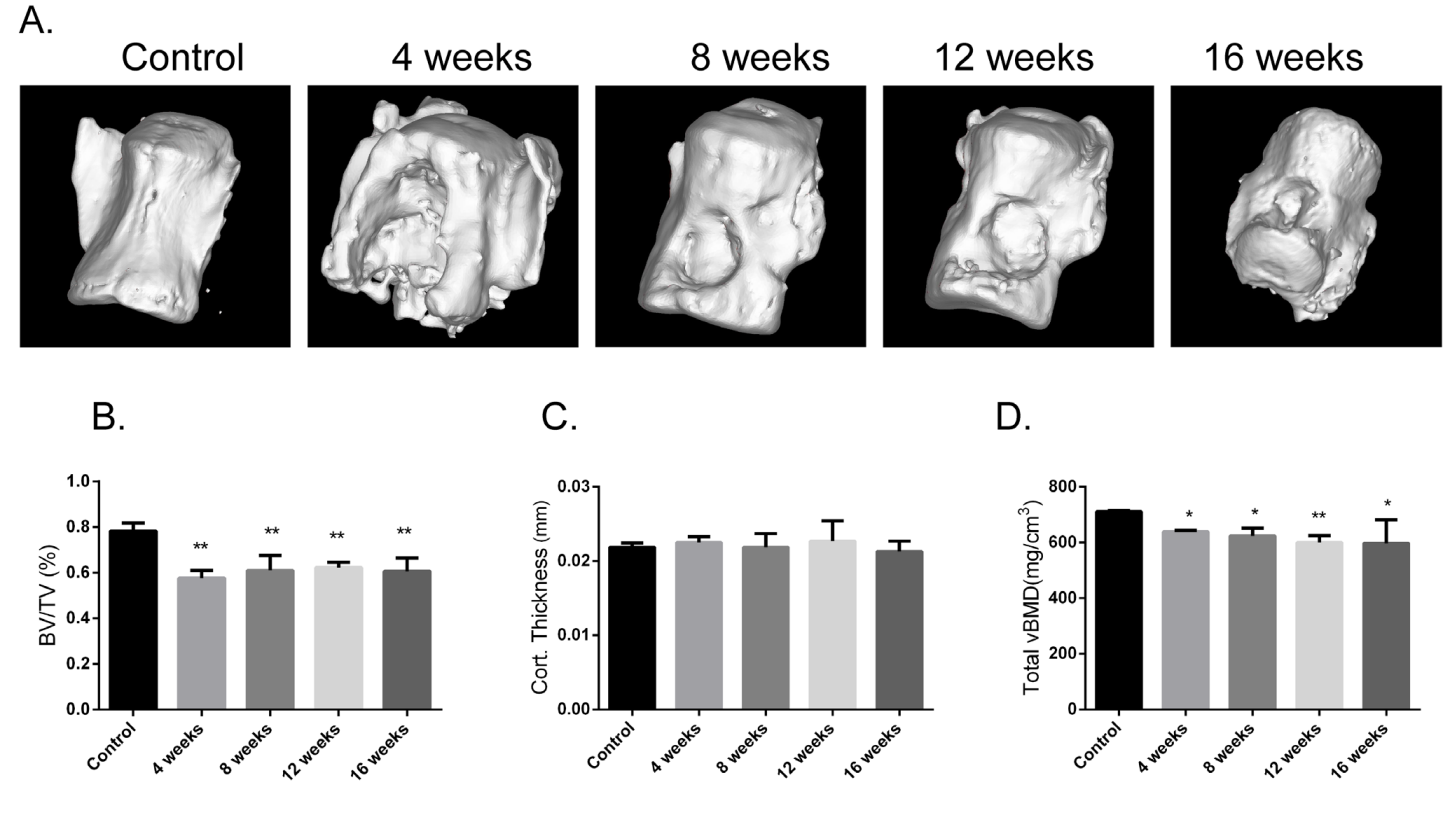
Assessment of bone microarchitecture Three-dimensional images assessment of L6s **A.** Assessment of BV/TV, cortical thickness, and total vBMD of L6s **B.**, **C.** and **D.** Values are the means ± SD. ^*^P < 0.05 *vs* the control group (*n* = 6).

The vertebral bone defect was successfully established in the rat model with an appropriate size, which was convincingly confirmed by bone microarchitecture detection. Compared to the control group, the model group showed significantly lower relative bone volume (BV/TV, %) and total volume BMD (vBMD, mg/cm^3^) at each time point (*P* < 0.05), while no significant differences in cortical thickness were observed for different time points. Further, in the model group, no significant differences in BV/TV, cortical thickness, or vBMD were observed for different time points. Three-dimensional images showed a significant bone defect in the model group at each time point, but no evidence of self-healing in the bone defect sites was observed after 16 weeks (Figure 4).

### Histological observation

**Figure 5:**
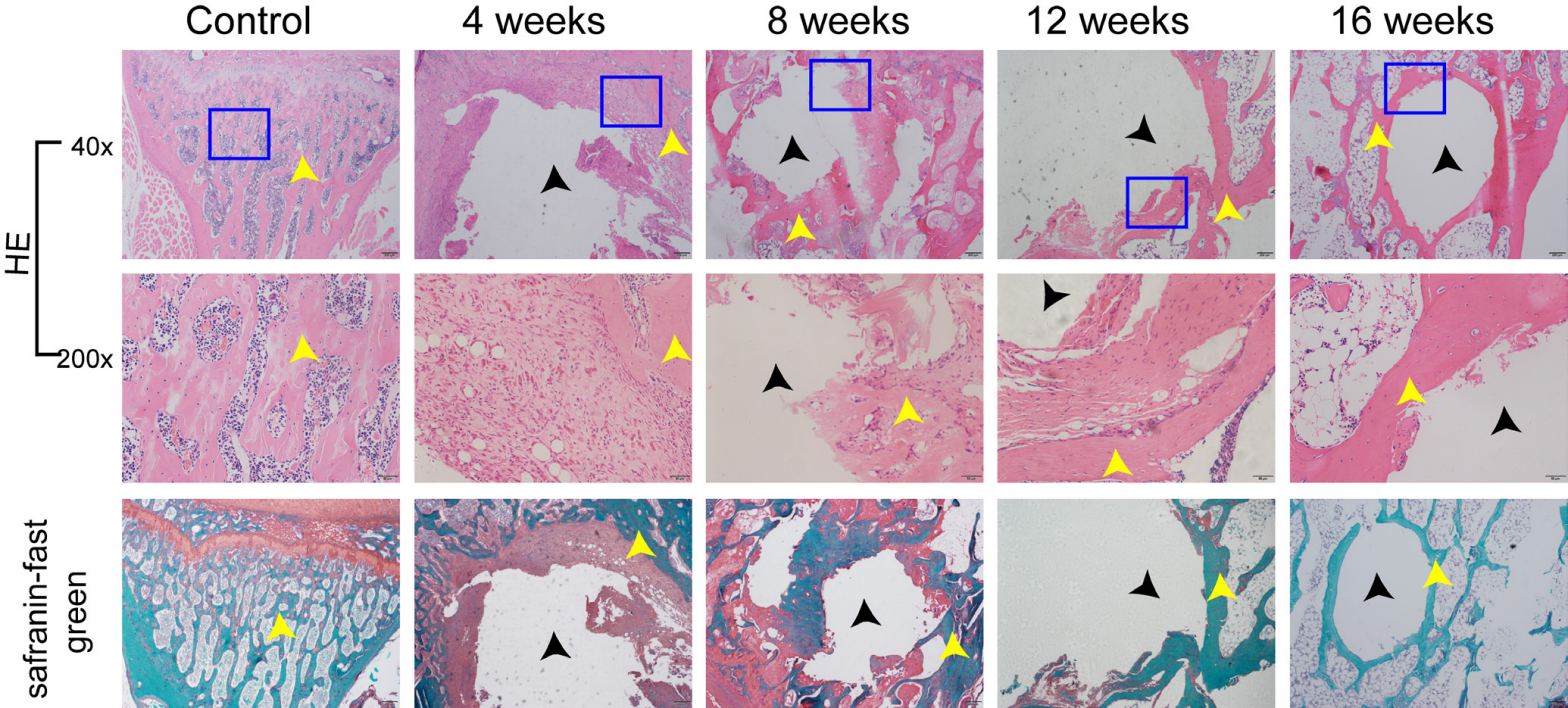
HE staining and Safranin-fast green staining were used for evaluating L6 histomorphometry The control group and the model group were compared as above. The black arrows stand for bone defect sites, and the yellow arrows stand for bone trabeculas. The images under high power field of microscope (200×) were placed above the images under low power field of microscope (40×).

Representative histological evidence of the bone defect from HE staining and Safranin-fast green staining further supported the findings obtained by micro-CT analysis. Unlike the control group, the model group showed a trabecular bone fracture sign and clear bone defects. Moreover, in the model group, no significant histological manifestation of self-healing could be observed among the different time points (Figure 5).

## DISCUSSION

Fragility fractures are associated with considerable disability and cost and an increased risk of mortality, which is particularly the case for fractures of the vertebrae [2]. Suitable animal models are the foundation for studies of OVF treatment; for instance, they could allow access to new biological materials that could promote fracture healing through vertebroplasty or kyphoplasty [14, 15]. Although previous reports described osteoporotic vertebral bone defect models in rabbits and sheep [9, 10], it is still necessary to develop additional feasible animal models, according to the FDA guidelines. Here, for the first time, we present a rat model of an osteoporotic vertebral bone defect that can sufficiently mimic the clinical occurrence of OVF.

For decades, studies have shown that the healing of defects in postmenopausal osteoporotic women is delayed [16-19]. As reported, at least 10% of OVF patients can be expected to experience a nonunion disease such as Kümmell’s disease [20], which is characterized by chest and back pain, a broken vertebral vacuum sign, vertebral collapse, and gradually progressive kyphosis after a few asymptomatic months; it may even cause spinal cord injury and paralysis [21, 22] due to gradual vertebral body collapse. In this study, we developed a rat model of an osteoporotic vertebral bone defect and observed no indication of self-healing after 16 weeks, suggesting that this rat model could commendably mimic patients with the osteoporotic vertebral nonunion reported in previous studies [23, 24].

At present, the two major pharmacological approaches to OP treatment and fracture prevention are anabolic agents that stimulate bone formation, such as parathyroid hormone, and anti-resorptive agents including bisphosphonates, calcitonin, raloxifene, and estrogen. However, clinically, a large proportion of patients with vertebral bone fracture or nonunion require combined treatment involving medical management and surgery to repair defects in osteoporotic vertebral bodies, stabilize the spine, and provide relief from pain. Although both vertebroplasty and kyphoplasty can alleviate pain successfully, they have potential disadvantages connected with the properties of polymethyl methacrylate, such as poor biocompatibility, no biological activity, no degradation and absorption properties, lacked direct bone apposition, thermal damage to surrounding tissues, overlarge inherent stiffness and elastic modulus that may increase the risk of fracture at adjacent spinal segments, and potential toxicity [25]. Luckily, more promising injectable bioactive cements with preferable biocompatibility and absorbability have been developed [26]. Many of these new cements with osteogenic effect have been evaluated in animal vertebrae, proximal tibia, or distal femur [27, 28]. However, outcomes obtained from these non-OP animal model studies cannot provide proof of this in osteoporotic vertebrae because the biomechanical environments are different. Although Zhang *et al.* [11] demonstrated the reinforcing effects of calcium sulfate cement bovine bone morphogenetic protein on vertebrae in a rabbit OP model, other animal models should be established to access the biomechanical factors of the new bioactive cement, such as osteoconductivity and osteoinductivity. Therefore, the rat model of osteoporotic vertebral bone defect developed in this study will play an important role in evaluating new bioactive bone substitutes in preclinical phases.

Selecting and establishing a proper animal model of osteoporotic vertebral bone defect is the precondition for studying OVF. In the present study, we developed an animal model of osteoporotic vertebral bone defect in 6-month-old rats to capitalize on the many advantages that rats have over other larger animals for laboratory studies. Laboratory-ovariectomized rats are an FDA-recommended model [29] to represent the process (e.g., cortical and trabecular bone loss) that occurs in the presence of cytokines or hormonal factors, owing to their fast generation at low cost, the fact that they are easy and safe to handle, their reliable reproducibility, and the similarities they have to human pathophysiological responses in postmenopausal cancellous bone loss [30, 31]. The choice of rat age (in months) is another important factor that should be considered. Skeletally immature rats achieve a low peak bone mass, which is considered as a high-risk factor for the development of human osteoporotic defects [32]. And to be honest, as for the quadruped animals, like rats, their spines are essentially loaded in the similar way with humans, and the axial compression force is reported to be higher, which contributes to the higher vertebral BMD [28]. In addition, because rats have been used in research for a long time, biological materials and techniques could easily be applied to this model. Accordingly, in our study, ovariectomized rats with vertebral bone defects were readily available for the simultaneous assessment of the therapeutic effects of anti-OP drugs and biomaterials, as well as other factors. Although the violation of the superior/inferior endplates in this animal model (a feature that is commonly present in OVFs) is absent, the protocol in this study is already a reasonable starting point that can be further modified, as required. This study is the first to describe the induction of an appropriately sized bone defect in the osteoporotic vertebral body. To sufficiently mimic OVF and nonunion and avoid the interference of spontaneous repair in the evaluation of bone repair with new biomaterials, the bone defect must be larger than this appropriate size [33]. In the present study, a 3 mm diameter hemispheric bone defect in the osteoporotic vertebral body developed via an anterior approach did not get completely self-repair by the end of the 16-week study, as revealed by sagittal plain X-rays, *ex vivo* naked-eye observations of L6, three-dimensional images of L6, and histological sections. We hypothesize that the nonunion of the osteoporotic vertebra reached a steady state retention period after 16 weeks. Overall, a 3 mm diameter hemispheric bone defect was therefore found to be an appropriately sized defect in the anterior part of the osteoporotic vertebral body in rats.

In conclusion, a rat model of osteoporotic vertebral bone defect, induced by OVX and an appropriately sized 3 mm diameter hemispheric vertebral bone defect made through surgery could sufficiently mimic OVF patients in clinic and provide a sound basis for subsequent studies. However, this rat model still has some disadvantages. For example, although the current rat model undermined the mechanical properties of the vertebrae, it cannot absolutely simulate the osteoporotic vertebral compressive fracture. More other animal models that can preferably simulate the osteoporotic vertebral compressive fracture using new techniques should be developed in the future. Simultaneously, due to the differences of anatomy and function between animal models and human, much more studies on vertebrae of animals and human cadavers should be developed to further confirm the applicability of the current model to OVF patients. In addition, the utility of the new bioactive bone substitutes, especially the injectable vertebral augmentation materials, applied to the current rat model, as well as the related pathologic changes and the molecule mechanism should be explored in future studies.

## MATERIALS AND METHODS

### Experimental animals

We used 6-month-old female Sprague-Dawley rats (n = 30). Rats were housed under conditions of controlled temperature (22-25°C) and constant atmospheric pressure (25 kPa) in an animal room under a 12 h light/dark cycle (lights on at 08:00). Food and water were available freely throughout the experiment. After an acclimation period of 1 week, rats were randomly assigned to one of two groups: those in the control group (n = 6) were euthanized at the start of the experiment (M0), and those in the model group (n = 24) were utilized in the development of the animal model with bilateral OVX and an appropriately sized bone defect in L6 and then were euthanized at the end of weeks 4, 8, 12, and 16.

### Ethical approval

All the experimental procedures were approved by the ethic committee of the First Affiliated Hospital of Guangzhou University of Chinese Medicine (license no., 20130425). Humane care was provided according to the Guidelines for the Care and Use of Laboratory Animals, published by the US National Institutes of Health.

### Surgical procedures

**Figure 6:**
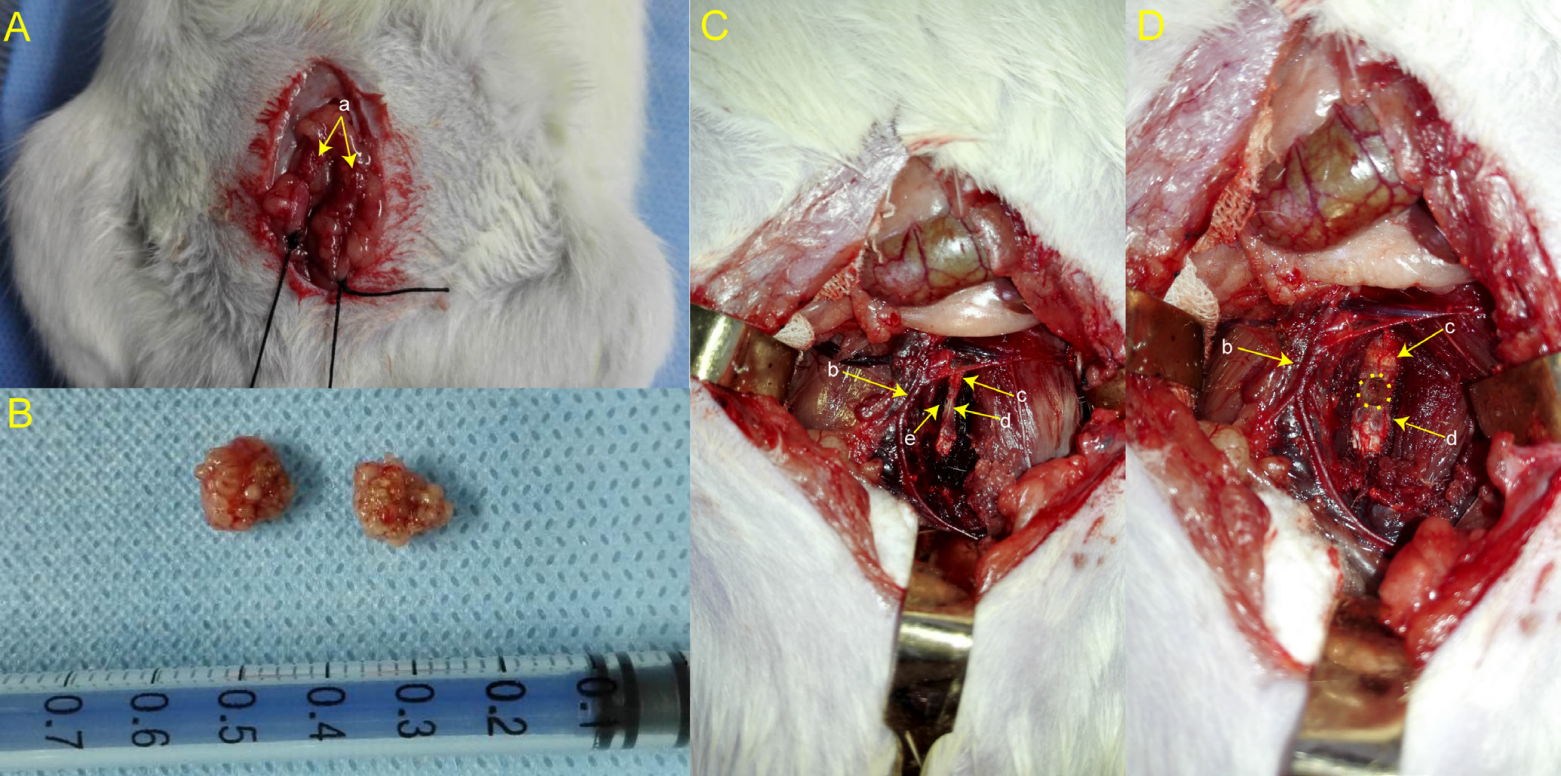
The bilateral ovaries were exposed during OVX **A.** The bilateral ovaries *in vitro* **B.** L6 was identified after separating a series of tissues **C.** A 3 mm diameter hemispheric bone defect was fabricated **D.** “a” represents ovary; “b” represents aorta abdominalis; “c” represents intervertebral disc; “d” represents vertebral body; “e” represents vertebral nourishing vessel entrance.

To induce an osteoporotic model, the rats in the model group were subjected to bilateral OVX (weight, 250 ± 10 g) according to the classic method reported in previous study [34] (Figure 6A and 6B). After anesthetization by intraperitoneal injection of pentobarbital sodium (3.0 mL/kg body weight), an abdominal incision was made around the midpoint between the lower margin of the free ribs and iliac crest, where the ovary is located. A suture was placed around the ovarian artery and vein prior to the removal of the ovary. To close the incision tightly, the muscles were repositioned in layers and sutured with resorbable sutures, and the skin was closed with nylon 4-0 sutures. The animals were given antibiotics postoperatively to prevent infection.

Rats in the model group were used to develop a vertebral bone defect 3 months after OVX. The animals were placed under general anesthesia by intraperitoneal injection (pentobarbital sodium 3.0 mL/kg body weight), and the target vertebra (L6) was exposed via an anterior midline transperitoneal approach. A linear skin incision of approximately 2 cm was made in the abdomen and blunt dissection of the muscles was performed to expose the enterocoelia. Most of the intestines were carefully moved to the side from the abdominal cavity and packed in a saline-soaked gauze pad during the surgical procedure. After separating the hind peritoneum and psoas major muscles and removing the aorta abdominalis to the side, the L6 was identified. The anterior longitudinal ligament and periosteum were removed by subperiosteal dissection to expose the L6 (Figure 6C). Then an appropriately sized bone defect (a 3 mm diameter hemispheric defect) was created through the anterior part of the vertebra using an electric drill with a 3 mm diameter drill bit at a slow speed and irrigated with a saline solution to avoid thermal necrosis. The drilled holes were rinsed by injection with a saline solution to remove bone fragments from the cavity (Figure 6D). Then the incision was closed in a layered fashion. The rats were maintained in the dorsal position prior to analepsia and received antibiotic therapy for 3 days after the operation. All of the animals were allowed to move freely around their cages following the operation.

### Sample preparation

At the start of the experiment (M0), rats from the control group (n = 6) were anesthetized for X-ray and DXA scanning and then were euthanized to acquire L6 samples. Three months after OVX, rats from the model group underwent bone defect surgery and then were anesthetized for X-ray and DXA scanning and euthanized to acquire L6 samples at the end of weeks 4 (n = 6), 8 (n = 6), 12 (n = 6), and 16 (n = 6) after surgery. L6 samples devoid of soft tissues were isolated and fixed in 4% phosphate-buffered paraformaldehyde for outside views, micro-CT analysis, and histological studies.

### Bone defect assessment

The vertebral bone defect site of L6 and the healing status of the bone defect were assessed using an X-ray scanner (AXIOM Aristos AX/TX/VX; Siemens, Germany). After anesthesia, rats were placed on the platform of the X-ray machine in the prone position. L6, the intersection of the bilateral iliac crest peak line and the spine, was defined as the center of the image. Sagittal plain X-ray images of the rats at M0, and weeks 4, 8, 12, and 16 were obtained at 50 kVp, 6.2 mAs, and 50 ms.

### Bone mineral content and density

The BMC (g) and BMD (g/cm^2^) of the rats were measured by DXA with a small-animal high-resolution collimator (Discovery A/SL/W/C; Hologic, Bedford, MA, USA). The rats were anaesthetized as described previously, and then were positioned on the DXA table in prone position with legs separated from the trunk to scan the whole bodies. After the scan, the regions of interest (ROIs) were marked across the entire L6 regions. Analyses were performed using the small-animal mode of the software supplied with the collimator (v. 13.2:3; Hologic), which was calibrated at the start of each experiment.

### Bone microarchitecture assessment

The L6s of all groups (n = 6 per group) were scanned using a small laboratory animal CT system (LaTheta LCT-200; Hitachi-Aloka, Tokyo, Japan), which was operated at 80 kV and 0.5 mA. All target vertebrae were placed in the 24 mm specimen holder to prevent movement. A whole bone scan was performed with an isotropic resolution of 192 μm^3^. A total of 180 slices with an isotropic resolution of 48 μm were collected. Thresholds for segmenting bone/soft tissue and trabecular/cortical bone were set at 160 and 500 mg/cm^3^, respectively, and the datasets were automatically segmented into the two compartments by LaTheta software (version 1.3). The microstructure of the cancellous bone was characterized using standard techniques to determine BV/TV (%), cortical thickness (mm), and vBMD (mg/cm^3^). Three-dimensional images were obtained through multiplanar reformation (Mimics 10.01).

### Histomorphometry

After micro-CT imaging, the L6 vertebrae, fixed in 4% phosphate-buffered paraformaldehyde for more than 24 h, were decalcified in 10% ethylene diaminetetraacetic acid (EDTA) for 4-6 weeks, with the solution changed twice per week, and then dehydrated by standard graded alcohol solutions, and embedded in LEICA Highmelt Paraffin (LEICA, Germany).To obtain a distinct view of the defect, the orientation and alignment of vertebrae were carefully considered during paraffin embedding. Bone tissues in paraffin were sectioned longitudinally into 5 µm thick. For general histological studies, HE staining and Safranin-fast green staining (LEICA, Germany) were performed according to manufacturer’s protocol. The slides were covered with neutral balsam (Shanghai, China, Batch No: 20141123) and observed under a microscope (BX53, OLYMPUS CORPORATION., Japan). The pictures were captured and analyzed with CellSens Dimension (Version 510-UMA-CellSens19.0-krishna-ch-00-01, Germany).

### Statistical analyses

All statistical analyses were performed using the software SPSS 19.0 (SPSS Inc, Chicago, IL, USA). All data were checked for normality and homogeneity of variance and are expressed as means ± standard deviation for the comparison of all parameters. Changes in BMD, BMC, and the microarchitecture at different time points were analyzed using a two-way analysis of variance (ANOVA). When significant differences were indicated by ANOVA, a pairwise comparison of time points was performed using the least significant difference test. In all analyses, *P* < 0.05 was taken to indicate statistical significance.

## Abbreviations

OVF: Osteoporotic Vertebral fractures
OP: Osteoporosis
VF: Vertebral fracture
OVX: Ovariectomy
BMD: Bone mineral density
FDA: Food and Drug Administration
BMC: Bone mineral content
DXA: Dual-energy X-ray absorptiometry
micro-CT: micro computed tomography
HE: Hematoxylin and eosin
L6: Lumbar vertebra 6
BV/TV: Relative bone volume
vBMD: Total volume BMD
ROIs: Regions of interest
EDTA: Ethylene diaminetetraacetic acid
